# Abstract of the Transactions of the American Medical Association

**Published:** 1864-06

**Authors:** 


### ABSTRACT OF THE TRANSACTIONS OF THE AMERICAN MEDICAL ASSOCIATION.

### *FIFTEENTH ANNUAL MEETING.*

Condensed from "New York Medical Independent."

#### FIRST DAY—MORNING SESSION.

At 11 o'clock A. M. order was called by the retiring President, Dr. ALLEN MARCH, of Albany, aided by Drs. J. COOPER, of Delaware, PRINCE, of Illinois, and COX, of the U. S. Volunteer Medical Staff.

Drs. JOHNTON and FURMAN, Secretaries, present.

All ex-Presidents and Secretaries were invited to a seat on the platform.

Dr. WILSON JEWELL, one of the ex-Presidents of this Society, was present, and took a seat upon the platform.

The roll of members by States, was then called by the Secretary.

Dr. BISSELL moved for a recess of ten minutes, to enable the delegates of the different States to appoint the Nominating Committee, which was carried.

The President then read his annual address, the subject of which was Medical Education.

On motion, it was resolved to thank the President for this able address, and to ask that a copy be furnished for publication.

Convention then adjourned until 3 o'clock.

It was announced that the following papers would be read:

1—By Dr. E. R. SQUIBB, a report on the Practical Working of the United States Drug Laws.

2—By the same, upon the same subject.

3—By Dr. C. C. COX, a report of American Medical Necrology.

4—By Dr. GURDON BUCK, a case of Plastic Operation.

5—By Dr. N. W. KINGSLEY, on Congenital Cleft; treatment of Congenital Fissure of Palate by mechanical appliances.

6—By Dr. J. H. GRISCOM, on the Physiological and Dietetic relations of Phosphorus.

7—By Dr. H. O. HITCHCOCK, on a Modified Ring Pessary.

8—By the same, Death from Air in the Circulation, introduced through Uterine Sinuses.

9—By Dr. ECHEVERIA, on Bromide of Potassa in Epilepsy, etc.

10—By Dr. DURHAMEL, on Spotted Fever in Washington, D. C.

11—By Dr. A. K. GARDNER, on Pessaries.

12—By Dr. L. WEBER, on Typhus and its treatment.

13—By Dr. J. KNEELAND, on Medicine and Surgery among the Onondaga Indians.

14—By Dr. C. W. PARSONS, on Medical Topography and Epidemics of Rhode Island.

### AFTERNOON SESSION.

Dr. S. G. ARMOUR, Chairman of the Committee on Nominations, presented the following report: For President, Dr. N. S. DAVIS, of Illinois. Vice Presidents, WM. H. MUSSEY, of Ohio; Dr. WORTHINGTON HOOKER, of Connecticut; Dr. WILLIAM WHEELAN, of District of Columbia; Dr. F. E. B. HINSEY, of Maryland. Treasurer, Dr. CASPER WISTAR, of Pennsylvania; Secretary, Dr. G. FURMAN, of New York. The next meeting of the Association is to be held in Boston.

Drs. ASKEW, of Del., and BISSELL, of N. Y., escorted the President elect to the chair. He said in substance, that he was very grateful for the honor conferred upon him. His interest in the profession was unabated. He had been identified with the As ociation from its incipiency, and was deeply interested in its progress. He regretted the absence of the Southern members of the Association, and hoped for their speedy return. The speech was brief, terse, conciliatory, and non-committal on the great questions of the time.

Invitations from various sources, extending courtesies to the delegates, were read and accepted.

Drs. PALMER, ASKEW and HUBBARD, were appointed by the Chair a Committee to examine "volunteer papers," and select those considered proper to read before that body.

Dr. GARDNER, of N. Y., spoke in relation to the appointment of a permanent Secretary.

Dr. HOOKER, of Conn., was in favor of a boiling-down process. He would abbreviate the amount of matter published. He did not see any necessity for a permanent Secretary, and was opposed to the incorporation of new regulations.

Dr. KENNEDY was in favor of paying the expenses of the Secretary.

Dr. MORGAN called for the settlement of the question without further debate.

Dr. LOOMIS was in favor of a permanent Secretary, a permanent Treasurer, and a permanent Committee of Five.

Dr. BOND, of Baltimore, thought it would be a proper time to decide upon the question when the money had been raised to pay the cost of such a luxury.

Dr. JEWELL argued in favor of a competent Secretary.

After considerable debate, the amendment in its original form (providing for a permanent Secretary to be elected for ten years,) was adopted. After which the meeting adjourned until Weddesday morning at 10 o'clock.

SECOND DAY,—MORNING SESSION.

Dr. DAVIS, the President, called the Association to order at the hour appointed for the commencement of the exercises.

The SECRETARY read the minutes of the last meeting.

Dr. C. W. STEARNS, of N. Y., was invited to participate in the proceedings of the meeting. Dr. KNIGHT, of Westchester Co., Dr. W. B. SOUTHARD, of Kalamazoo, Mich., Dr. O. WHITE, of Oswego, and Dr. LIVINGSTON, of Mass., were also elected members by invitation, and allowed to take part in the proceedings. Dr. BROWN SEQUARD, of Boston, and Dr. JOHN P. GRAY, of the State Lunatic Asylum, Utica, were elected permanent members of the Association. The former, on being introduced to the Association, made a brief and modest acknowledgment of the honor conferred upon him.

Dr. WISTAR, the Treasurer, reported that the demand for copies of the annual volume was exceedingly small, only 150 copies having been sold, though the Society numbers 3,000 members. The receipts of the year amount to $943; balance on hand $450.

The following white-haired and venerable gentlemen were introduced as seniors in the profession: Dr. GREEN, Mass., Dr. COCK, N. H., Dr. NOYES, Mass., Dr. PARSONS, Conn., and Dr. GERBERT, Phila., were invited to take seats upon the platform. The regular order of business was resumed, and reports were called for.

Upon a re consideration of the vote relative to the report of the Committee on Compulsory Vaccination, that report was read. The Committee, consisting of Drs. JAMES F. HIBBARD, WILSON JEWELL, and JOHN H. GRISCOM, report that, for various reasons, they deem compulsory vaccination impracticable, and recommend the following resolutions:

*Resolved*, That this Association deems it its duty to institute measures looking to the vaccination of every person within the limits of country over which it exercises jurisdiction.

*Resolved*, That a Committee be appointed under the direction of this Association, to take cognizance of all matters pertaining to vaccination.

*Resolved*, That a Committee of —— be appointed in each State, to superintend the measure in its State, which Committee shall be subordinate, auxiliary, and advisory to the Central Committee.

The report was referred to Section on Hygiene.

The most important part of the proceedings to-day was a set of resolutions offered by Prof. GARDNER, requesting Congress to enact a law which will not classify drugs and medicines among the contrabands of war, so that horrors of war may be mitigated in the South as well as in the North, and asking the government to permit a flag of truce boat to go South with medicines to be applied for the benefit of wounded Union and rebel soldiers in rebel hospitals, who, from the exigencies of war, cannot be properly treated by the surgeons of the rebel army. In offering the resolutions, Dr. GARDNER said he was actuated by motives of common humanity, and for the advancement of medical science. He hoped they would be adopted.

The election of permanent Secretary was made the order of the day for Thursday.

A vote of thanks to the retiring President, ALDEN MARCH, M. D., was passed.

Dr. C. C. Cox, medical purveyor of Baltimore, addressed the Convention upon the subject of the acknowledgment due to members of the profession who are serving their country in the army.

Dr. HAMILTON of New York, and Dr. MUSSEY of Chicago, spoke in support of the resolution, which was subsequently adopted.

[*To be concluded in July number.*]

